# New Cytotoxic Seco-Type Triterpene and Labdane-Type Diterpenes from *Nuxia oppositifolia*

**DOI:** 10.3390/molecules22030389

**Published:** 2017-03-02

**Authors:** Shaza M. Al-Massarani, Ali A. El-Gamal, Mohammad K. Parvez, Mohammed S. Al-Dosari, Mansour S. Al-Said, Maged S. Abdel-Kader, Omer A. Basudan

**Affiliations:** 1 Department of Pharmacognosy, College of Pharmacy, King Saud University, Riyadh 11451, Saudi Arabia; salmassarani@ksu.edu.sa (S.M.A.-M.); khalid_parvez@yahoo.com (M.K.P.); mdosari@ksu.edu.sa (M.S.A.-D.); msalsaid@ksu.edu.sa (M.S.A.-S.); basudan@ksu.edu.sa (O.A.B.); 2 Department of Pharmacognosy, Faculty of Pharmacy, Mansoura University, El-Mansoura 35516, Egypt; 3 Department of Pharmacognosy, College of Pharmacy, Prince Sattam bin Abdulaziz University, Al-kharj 11942, Saudi Arabia; m.youssef@psau.edu.sa; 4 Department of Pharmacognosy, College of Pharmacy, Alexandria University, Alexandria 21215, Egypt

**Keywords:** *Nuxia oppositifolia*, labdane-type diterpene, seco-triterpene, triterpenes, cytotoxicity

## Abstract

Chromatographic purification of the *n*-hexane and dichloromethane extracts of *Nuxia oppositifolia* aerial parts, growing in Saudi Arabia, resulted in the isolation and characterization of three new labdane-type diterpene acids, 2β-acetoxy-labda-7-en-15-oic acid (**1**), 2β-acetoxy-7-oxolabda-8-en-15-oic acid (**2**), 2β-acetoxy-6-oxolabda-7-en-15-oic acid (**3**), and one new seco-triterpene, 3,4-seco olean-12-en-3,30 dioic acid (**4**), together with 10 known lupane, oleanane and ursane-type triterpenes, as well as the common phytosterols, β-sitosterol and stigmasterol (**5**–**16**). Their structures have been assigned on the basis of different spectroscopic techniques including 1D and 2D NMR. Moreover, 13 of the isolated compounds were tested on the human cancer cell lines HeLa (cervical), A549 (lung) and MDA (breast), and most of the compounds showed potent cytotoxic activities in vitro.

## 1. Introduction

The genus *Nuxia* (family Buddlejaceae) comprises ca. 40 species of shrubs and trees distributed over the southern region of the Arabian peninsula, tropical Africa (Madagascar, Comoro and the Mascarene Islands) and South Africa [1]. *Nuxia* is represented in Saudi Arabia by only two species, viz. *N. oppositifolia* Benth and *N. congesta* Fresen, with limited distribution in the southwestern parts of the Hijaz area [2,3]. Several *Nuxia* species are plants of economic and medicinal interest with a rich diversity of ethnobotanical uses. Some species are used for the treatment of urine albumin, venereal disease, and as purgatives [4]. Leaves of *N. sphaerocephala* Baker are used in the traditional medicine of Madagascar to treat malarial splenomegaly and infantile hydrocephalus [5].

The dichloromethane leaf extract of *N. verticillata*, endemic to the Mascarene Archipelago, had potential anti-proliferative activity and selectively inhibited cancer cell growth in comparison with normal cells [4]. Some of the *Nuxia* plants have been studied phytochemically, revealing the presence of phenylpropanoid glycosides such as verbascoside [6], in addition to clerodane and labdane diterpenoids, and pentacyclic triterpenes [5].

Recently, various studies have shown pentacyclic triterpenes as valuable candidates to treat cancer by several modes of action [7]. Reviewing literature revealed that few phytochemical and biological studies have been conducted on *N. oppositifolia*, where only two compounds, namely verbascoside and the acylated diglycoside 2′′-acetyl-3′′-benzoyl-nuxioside, have been reported [6] These facts encouraged us to reinvestigate the chemical constituents and biological activities of *N. oppositifolia*, aiming to find new entities able to cure contemporary diseases such as cancer. In this paper, we describe the isolation and structural elucidation of four new compounds and 11 known compounds as well as assessment of in vitro cytotoxic activities of some of the isolated compounds against three cancer cell lines.

## 2. Results and Discussion

### 2.1. Phytochemical Study

**Figure 1:**
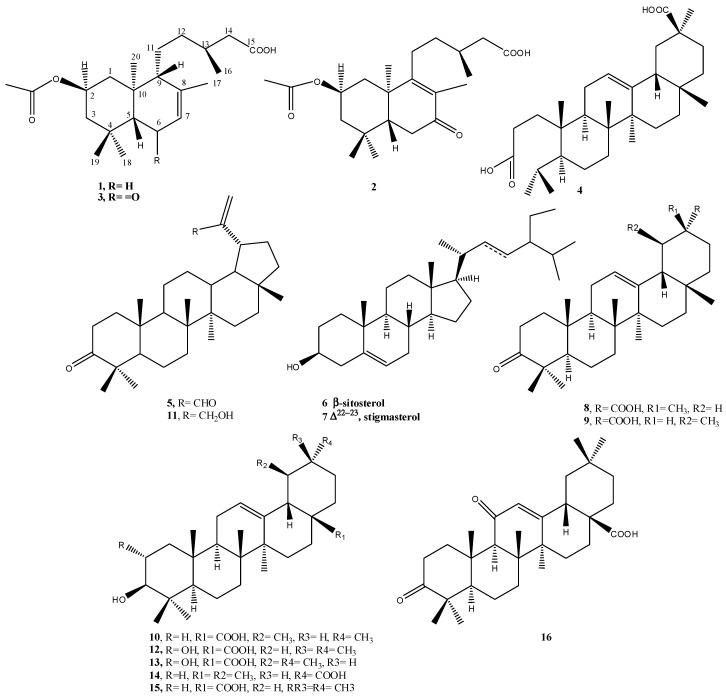
Structures of isolated compounds from aerial parts of *N. oppositifolia.*

As part of our search for novel medicinal compounds from plants, we investigated the aerial parts of *N. oppositifolia*. Chromatographic separation and purification resulted in the isolation and full identification of three new labdane diterpenoic acids (**1**–**3**), a new seco-triterpene, 3,4-seco olean-12-en-3,30 dioic acid (**4**), along with nine known triterpenes, and the common phytosterols β-sitosterol and stigmasterol (**5**–**16**) (Figure 1).

**Figure 2:**
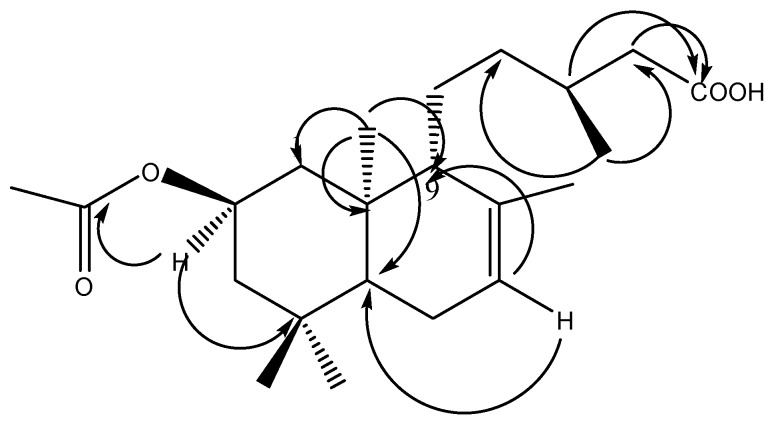
Key HMBC correlations of compound **1**.

The molecular formula of compound **1**, obtained as a viscous liquid, was determined to be C_22_H_36_O_4_ by HR-EI-MS analysis (*m*/*z* 364.2614 [M^+^]) with five degrees of unsaturation. IR showed intense absorption bands at 3470 (OH), 1745 (CO_2_R), 1715 (CO_2_H) and 1665 (C=C), suggesting the presence of OH, two carbonyl groups assigned to an ester and free carboxylic acid groups and an olefinic double bond, respectively. The carbonyl groups showed signals at δ_C_ 170.8 and 179.0 ppm in ^13^C-NMR. ^1^H-NMR revealed the presence of six methyl groups at δ_H_ 2.00, 1.62, 0.93, 0.92, 0.87 and 0.79 ppm, assigned to acetate, olefinic (Me-17), secondary methyl (Me-16), and three tertiary methyl groups, Me-19, Me-18 and Me-20, respectively. The presence of a trisubstituted double bond was evident from an olefinic methyl group at δ_H_ 1.62 ppm allylically coupled to the olefinic proton at δ_H_ 5.34 (H-5). Additionally, the ^1^H-NMR spectra showed a downfield triplet centered at 4.95 ppm and correlated it directly to a carbon signal at δ_C_ 68.9 in an HSQC experiment, and assigned it to H-2. The ^13^C-NMR and distortionless enhancement by polarization transfer (DEPT) experiment afforded a total of 22 carbon atoms ascribed to six methyl, six methylene, five methine groups and five quaternary carbon atoms, two of them appearing at δ_C_ 170.8 and 179.0 ppm, assigned to an acetate carbonyl at C-2 and a free carboxylic acid carbonyl at C15. Based on the above spectral evidence, the structure of (**1**) was proposed to be a labdane-type diterpene with an acetoxyl group, a trisubstituted double bond and a free carboxylic acid group [5]. The ^1^H-^1^H COSY experiment showed cross-peak correlations between the downfield proton at δ_H_ 4.94 (H-2) and both protons at positions H-1 (α and β), H-2 (α and β) at δ_H_ 1.00, 2.13, 1.17 and 1.71, respectively, positioning the acetoxyl group at C-2. An additional COSY correlation was detected between the secondary methyl at δ_H_ 0.93 (Me-16) and the multiplet proton at δ_H_ 1.80 (H-13). Moreover, it showed a correlation between the broad olefinic proton at δ_H_ 5.34 (H-7) and both signals at δ_H_ 1.90 (H-6) and 1.62 (Me-17). The exact positions for the acetoxyl at C-2 and the free carboxylic acid groups at C-14 were proved through interpretation of the HMBC experiment where significant ^2,3^*J* correlations were observed from the methine proton at 4.94 ppm (H-2) and the acetoxyl carbonyl carbon at 170.8 ppm; from Me-16 protons at 0.93 ppm to C-12 and C-14; and from the methylene protons at 1.80, 2.07 (H-13, H-14) and the free carbonyl of the carboxylic acid group at C-15 (δ_C_ 179.0). Finally, the location of the double bond at C-7 was confirmed, also from the HMBC experiment, showing two and three bond correlations from the olefinic proton at δ_H_ 5.34 (C-7) to both C-5 and C-9 at 49.5 and 54.3 ppm, respectively (Figure 2). The relative stereochemistry was determined by the NOESY experiment where significant cross-peaks were observed between Me-20, Me-18 and H-2, demonstrating that all are on the lower face of the molecule (α-form), while Me-19, H-5, H-9 and Me-16 are on the opposite side of the molecule (β- form). Compound **1**, identified as 2β-acetoxy-labda-7-en-15-oic acid, is reported here for the first time from a natural source. To the best of our knowledge, the deacetylated derivative of compound **1**, 2α-hydroxylabda-7-en-15 oic acid, has been isolated before from the aerial parts of *Brickellia lanciniata* [8].

Compound **2**, obtained as a viscous liquid, showed in HR-EI-MS [M^+^] 378.2406 (calcd. for 378.2400, C_22_H_34_O_5_). The spectral data of compound **2** showed, like compound **1**, the skeleton of a labdane-type diterpene [5]. IR and UV suggested the presence of an α,β-unsaturated carbonyl group (IR 1662 cm^−1^, UV 246 nm). The ^1^H- and ^13^C-NMR results (Table 1 and Table 2) also showed spectral data similar to **1** with six methyl groups, one assigned to acetate and the other five assigned to the labdane diterpene skeleton, distinguished into one secondary methyl group at δ_H_ 0.95 (d, *J* = 6.2 Hz) and four tertiary methyl groups, one of which is an olefinic methyl appearing as a singlet at δ_H_ 1.64. It showed also, like compound **1**, an acetoxyl group located at position 2, a downfield proton centered at 5.00 ppm (H-2). The absence of an olefinic proton in the ^1^H-NMR spectrum and the presence of two downfield quaternary carbons at δ_C_ 130.0 (C-8) and 167.1 (C-9) proved the existence of a tetrasubstituted double bond conjugated with a carbonyl carbon at δ_C_ 199.0 (C-7) (α,β-unsaturated double bond). The significant cross-peak correlations observed in ^1^H-^1^H COSY between both H-1 (δ_H_ 1.27 and 1.18) and H-2 (δ_H_ 4.98) were also clear in compound **2** and confirmed the position of the acetoxyl group at C-2. The main difference between **1** and **2** was observed in the ^13^C-NMR and DEPT experiment where the number of methine carbons in compound **2** was less by two (three in **2** instead of five in **1**), while the number of quaternary carbon atoms increased to six in **2** vs. five in **1**. The HMBC experiment verified the exact structure of compound **2** as 2β-acetoxy-7-oxolabda-8-en-15-oic acid, through two- and three-bond correlations observed from olefinic methyl (Me-17) at δ_H_ 1.64 to C-9 (δ_C_ 167.0), C-7 (δ_C_ 199.9) and C-8 (δ_C_ 130.0); from Me-20 protons (δ_H_ 1.06) to C-5 (δ_C_ 49.5) and C-9 (δ_C_ 130.0); from both α and β methylene protons at C-11 (δ_H_ 2.03, 2.19) to C-8 and C-9; from Me-16 (δ_H_ 0.95) to C-12 (δ_C_ 35.2) and C-14 (δ_C_ 40.9); and finally from H-13 at δ_H_ 1.95 to C-14 (δ_C_ 41.5) and C-15 (δ_C_ 177.9). Like compound **1**, the relative stereochemistry was determined by interpretation of the NOESY experiment. Compound **2** is reported here for the first time from a natural source.

Compound **3**, isolated as a viscous liquid, showed the same [M^+^] 378.2406 in HR-EI-MS as **2**. The presence of a carbonyl of unsaturated ketone was suggested by intense IR absorption bands at 1737, 1662 cm^−1^ and UV_λmax_ at 246 nm. NMR data showed, like compounds **1** and **2**, the basic skeleton of labdane-type diterpenes [5], also with an acetate moiety, a free carboxylic acid and an unsaturated ketone (conjugated with an olefinic double bond) observed at δ_C_ 170.7, 177.9 and 199.1 ppm in ^13^C-NMR.

The ^13^C-NMR and DEPT experiments showed a close resemblance to **1** where a total of 22 carbon atoms were counted and ascribed to six methyl, five methylene, five methine groups, and six quaternary carbon atoms. The above data revealed that **3** is an analogue to compound **1**, except that the number of methylene carbons was decreased by one and subsequently the number of quaternary carbons was increased by 1. The rest of the NMR data were closely similar to **1**. The ^1^H-NMR showed, also like **1**, the presence of a singlet olefinic proton at δ_H_ 5.70 assigned to H-7, a downfield oxygenated proton at 4.90 ppm assigned to H-2, a singlet olefinic methyl at 1.82 ppm assigned to Me-17 with allylic coupling in the COSY experiment, with the olefinic proton at C-7. A significant singlet signal appeared at δ_H_ 1.97, integrated with one proton and assigned to H-5. Comparing the chemical shift and the splitting pattern of H-5 in **3** with that of **1** pointed to the existence of a nearby electron-withdrawing group (CO at C-6) in compound **3**. The HMBC experiment was able to verify the structure of **3** as 2β-acetoxy-labda-7-ene-6-oxo-15 oic acid by clear two- and three-bond cross-peaks observed from Me-17 at δ_H_ 1.82 to C-7 (128.5 ppm) and C-8 (158.9 ppm); from H-7 at δ_H_ 5.70 to C-5 (62.5 ppm) and C-9 (56.4 ppm); from H-1 at δ_H_ 2.03 to the acetate carbonyl (170.7 ppm); from H-3 at δ_H_ 1.61 to C-2 (67.9 ppm); from Me-16 to C-12 (38.9 ppm) and C-14 (41.5); from H-13 at δ_H_ 1.93 to C-15 (177.9 ppm); and finally from Me-20 at δ_H_ 0.82 to C-5, C-9 and C-10. Additionally, the 2D NOESY experiment was useful for the determination of the relative stereochemistry of **3** where significant cross-peaks were observed between Me-18, M-20 and H-2, demonstrating that all are on the lower face of the molecule, while Me-19, H-5 and Me-16 are on the opposite side of the molecule (β-form). Therefore, the final structure of **3** was established as 2β-acetoxy-6-oxolabda-7-en-15-oic acid, isolated here for the first time from natural source as an isomer of **2**.

**Figure 3:**
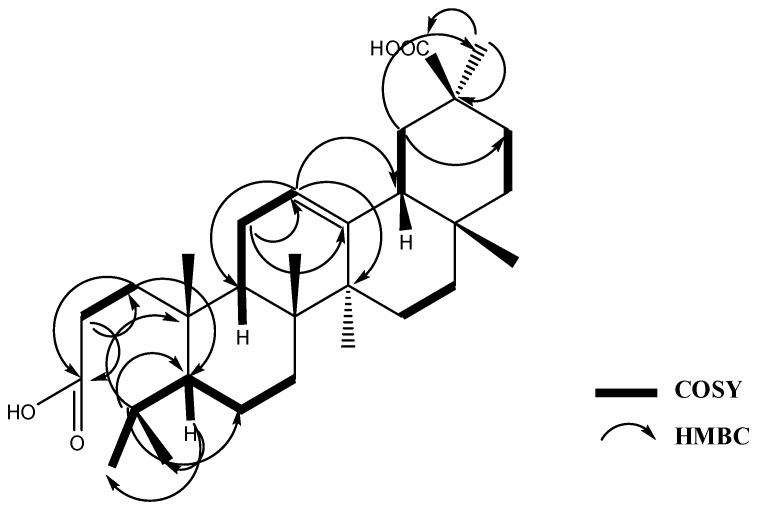
Key HMBC and COSY correlations of compound **4**.

Compound **4**, isolated as a white powder, had the molecular formula C_30_H_48_O_4_ as deduced from HR-EI-MS *m*/*z* 472.3553 [M^+^] (calcd. for 472.3553). The basic skeleton of **4** was proved to be a triterpene based on the assignment of the data obtained from MS and NMR. HR-EI-MS showed a molecular ion peak at *m*/*z* at 472 calculated for C_30_H_48_O_4_ with seven degrees of unsaturation, three of which were accounted for by one olefinic and two carbonyls of free carboxylic acids; the rest of the unsaturation number were allocated to four cyclic rings, indicating that **4** is a seco pentacyclic triterpene with two carboxylic acid moieties. Characteristic fragments were observed in EI-MS at *m*/*z* 248, 238 and 203, suggesting the presence of an oleanane-type triterpene with a double bond at C-12 (∆^12^). The presence of a significant fragment at *m*/*z* 248 is an explicit pattern of fragmentation characteristic of oleanane-type triterpenes conforming to rings D and E [9,10] while the fragments at *m*/*z* 233 and 203 were due to the loss of the methyl and carboxylic acid groups from the fragment at *m*/*z* 248. The ^1^H-NMR results showed that a broad singlet olefinic proton appeared at δ_H_ 5.19 (brs, H-12), five tertiary methyl groups (Me-25, 26, 27, 28 and 29) and two secondary methyl groups (Me-23 and 24); the latter were coupled to protons at δ_H_ 1.71 (m, H-4) and 0.96 (brd, *J* = 11.7, H-5) in the COSY experiment (Figure 3), indicating the presence of a separate isopropyl moiety at C-5. A total of 30 carbons were observed in the ^13^C-NMR spectrum, differentiated into seven methyl, 10 methylene, five methane groups and eight quaternary carbon atoms with the aid of the HSQC and DEPT experiments. Three downfield quaternary carbon atoms appeared at δ_C_ 174.8, 177.9 and 144.2 ppm, assigned to C-3, C-30 and C-13. The remaining quaternary carbon atoms resonated at δ_C_ 31.6, 39.0, 39.5, 41.6 and 43.1, assigned to carbon atoms 17, 8, 10, 14 and 20, respectively. The above data proved the presence of a ∆^12^ seco oleanan–type triterpene with two free carboxylic acid groups at C-3 and C-30. The HMBC experiment revealed two- and three-bond correlations observed from H-1 methylene protons at δ_H_ 1.54 to C-3, C-5; from H-12 (δ_H_ 5.19) to C-9, C-14 and C-18; from H-2 at 2.04 ppm to C-1 and C-3; from H-11 at δ_H_ 1.80 m to C-12 and C-13; from Me-29 protons at δ_H_ 1.04 to C-20 and C-30; from H-19 at δ_H_ 1.59 (brd, *J* = 13.4) to C-21 and C-29; from H-5 to C-23 and C-24; and finally from H-4 to C-5, C-6 and C-10. The latter correlations proved the attachment of the isopropyl moiety at C-5 (Figure 3). The upfield shift of the methyl protons at 1.06 (Me-29) verified the location of the second free carboxylic acid group to be at C-30 [11]. The presence of the free carboxylic acid group at C-3 and the isopropyl moiety at C-5 verified that compound **4** is a new 3,4-seco oleanane triterpene and its final structure is 3,4-seco-olean-12 ene-3,30 dioic acid. It is worth mentioning that an isomer of compound **4** has been isolated from *Junellia tridens* [9], where the locations of the dicarboxylic acid groups were at positions 3 and 28 rather than 3 and 30 as in **4**.

The known compounds were identified as 3-oxolup-20(29)-en-30-al (3-oxolupenal) (**5**) [12,13], β-sitosterol and stigmasterol (**6**, **7**), 3-oxoolean-12-en-29α-oic acid (katononic acid) (**8**) [14,15], urs-12-en-3-one-30-oic acid (ifflaionic acid) (**9**) [16,17], ursolic acid (**10**) [5] 30-hydroxylup-20(29)-en-3-one (3-oxolupenol) (**11**) [12,18], 2α,3β-dihydroxyolean-12-ene-28-oic acid (maslinic acid) (**12**), 2α-hydroxy-ursolic acid (asiatic acid) (**13**) [19], 3-hydroxyurs-12-en-30-oic acid (plectranthoic acid A) (**14**) [20], oleanolic acid (**15**) [21], and 3,11-dioxoolean-12-en-28-oic acid (**16**) [22]. The identities of the known compounds were determined by analysis of their spectroscopic data and comparison with those reported in the literature.

Notably, all isolated compounds, except **6**, **10**, **11** and **15**, are reported here from the genus *Nuxia* for the first time.

### 2.2. Cytotoxicity Assay

Most of the isolated compounds were evaluated for their in vitro cytotoxic activities against human cervical (HeLa), lung (A549) and breast (MDA) cancer cell lines. Among these, compound **8** possessed the most significant activity (IC_50_ = 29.35 μg/mL) against HeLa cells, while compound **4** showed the highest cytotoxic potential against A549 cells (IC_50_ = 53.78 μg/mL) (Table 3).

## 3. Materials and Methods

### 3.1. General Procedures

A high-resolution mass spectrophotometer, Jeol JMS-700, was used for accurate mass determination with electron impact mode of ionization at 70 ev. Direct probe was used with temperature ramp setting, initial temperature 50 °C rise, rate of 32 °C per minute and final temperature set up to 350 °C. The IR spectra were recorded on JASCO 320-A spectrometers, optical activity was measured on Jasco P-2000 Polarimeter, Japan while the ^1^H- and ^13^C-NMR spectra were recorded at the NMR Unit at the College of Pharmacy, Prince Sattam Bin Abdulaziz University, on an Ultra Shield Plus 500 MHz (Bruker, Billerica, MA, USA) spectrometer operating at 500 MHz for proton and 125 MHz for carbon, respectively. The chemical shift values are reported in δ (ppm) relative to the internal standard TMS; the coupling constants (*J*) are reported in Hertz (Hz). The 2D NMR experiments (COSY, HSQC, HMBC, and NOESY) were obtained using a standard Bruker program.

Isolations of compounds was performed using Silica gel 60 (0.063–0.200 mm, Merck, Darmstadt Germany) and Sephadex LH-20 (Fluka, Buchs, Switzerland) for open column chromatographic separations, while Lichroprep RP-18 (25–40 µm, Merck) reversed phase material was used for vacuum liquid chromatography (VLC). Centrifugal preparative thin layer chromatography (CPTLC) was performed on a Chromatotron device (Harrison Research, Palo Alto, CA, USA). Plates coated with 1 and 2 mm of silica gel 60, 0.04–0.06 mm were used. All solvents used were of analytical grade. TLC was performed on pre-coated silica gel F254 plates (E. Merck, Darmstadt, Germany); detection performed by spraying with *p*-anisaldehyde/H_2_SO_4_ reagent at 254 nm. All chemicals were purchased from Sigma Chemical Company (St. Louis, MO, USA). The absorbance was read on a microplate reader (ELX 800, Bio-Tek Instruments, Winooski, VT, USA) at 549 nm.

### 3.2. Plant Material

Aerial part (leaves, stems and flowers) of *N. oppositifolia* was collected from Wadi Lajab in Jazan province of Saudi Arabia in March 2012 and identified at the Pharmacognosy Department, College of Pharmacy, King Saud University. A voucher specimen (Voucher # 15501) was deposited at the Pharmacognosy Department, College of Pharmacy, King Saud University.

### 3.3. Extraction and Isolation

The dried and powdered aerial parts of *N. oppositifolia* (900 g) were extracted by maceration with 80% ethanol (4 × 2 L) at room temperature. The combined obtained ethanolic extract was filtered and concentrated under reduced pressure at 40 °C using a rotary evaporator. The dried ethanolic extract (105 g) was redissolved in 40% ethanol and successively partitioned for several times with *n*-hexane (3 × 500 mL), chloroform (3 × 500 mL) and *n*-butanol (3 × 500 mL) to provide the corresponding extracts.

The *n*-hexane fraction (17.6 g) was subjected to column chromatography on pre-packed silica gel column (40 mm i.d. × 350 mm) and eluted with *n*-hexane-ethyl acetate gradient. Collected fractions were examined with TLC and similar ones were pooled together into four fractions (A–D). Fraction A, eluted with 5% EtOAc/*n*-hexane, was further purified with a Chromatotron device (0.5% EtOAc/CHCl_3_, 2 mm) to yield pure compounds **5** (170 mg), **6** and **7**. Part of fraction B, eluted with 10% EtOAc/*n*-hexane, afforded upon crystallization, compounds **8**, **9** and 15 in pure form, whereas another part of the same fraction gave compound **1** upon further purification using RP-18 CC with 5% H_2_O/MeOH. Fraction C eluted with 20% EtOAc/*n*-hexane, afforded compound **10** after solvent evaporation, while subsequent centrifugal purification with chromatotron using 25% EtOAc/*n*-hexane provided compound **11** in a clear form. Fraction D eluted with 30% EtOAc/*n*-hexane afforded two main subtractions Da, Db. Subfraction Da was subjected to RP-18 CC using 5% H_2_O/MeOH to afford compounds **13** and **14,** while elution with 10% H_2_O/MeOH gave compound **12**. Subfraction Db was applied to Centrifugal radial TLC using 0.5% MeOH/CHCl_3_ to afford compound **4**. The chloroform fraction (8 g) was loaded on a silica gel CC and eluted with increasing amounts of MeOH/CHCl_3_ yielding four major fractions (E–H). The fraction eluted with 1% MeOH/CHCl_3_ (E) was subjected to sephadex LH-20 column to afford sub-fractions a–c. Subsequent purification of the sub-fraction a by RP-18 CC (MeOH/H_2_O, 90:10) yielded compound **15** (5 mg) directly in pure form, while compounds **2** and **3** were obtained in pure form after further chromatotron purification with 7% ethanol in *n*-hexane.

### 3.4. Spectral Data of New Compounds

*2*β*-Acetoxy-labda-7-ene-15(E)-oic acid* (**1**), 80 mg; viscous; ^1^H- and ^13^C-NMR: see Table 1 and Table 2; [α]_D_ = −7.0, (CHCl_3_), IR (KBr) ν_max_ cm^−1^: 3470, 2960, 1745, 1715, 1665, 1463, 1380, 1245, 1027; HREIMS: *m*/*z* = 364.2614 calc. for C_22_H_36_O_4_.

*2*β*-Acetoxy-7-oxolabda-8-ene-15(E)-oic acid* (**2**), 70 mg; viscous; ^1^H- and ^13^C-NMR: see Table 1 and Table 2; [α]_D_ = 0, (CHCl_3_), IR (KBr)ν_max_ cm^−1^: 3450, 2960, 29232873, 1733 (O-C=O), 1662. 1465, 1377, 1161, 1050; HREIMS: *m*/*z* = 378.2406 calc. for C_22_H_34_O_5_.

*2*β*-Acetoxy-6-oxolabda-7-ene-15(E)-oic acid* (**3**), 70 mg; viscous liquid; ^1^H- and ^13^C-NMR: see Table 1 and Table 2; [α]_D_ = 0, (CHCl_3_), IR (KBr) ν_max_ cm^−1^: 3500, 1737, 1662, 1465, 1370, 1245, 1029; HREIMS: *m*/*z* = 378.2406 calc. for C_22_H_34_O_5_.

*3,4-Seco olean-12-en-3,30 dioic acid* (**4**), 12 mg, C_30_H_48_O_4_; solid; ^1^H- and ^13^C-NMR: see Table 1 and Table 2; [α]_D_ = +15 (MeOH), IR (KBr)ν_max_ cm^−1^, 3210, 1700 (COOH), 1640 (C=C), 1212, 960 cm; HREIMS: *m*/*z* = 472.3553 calc. for C_30_H_44_O_4_.

### 3.5. Spectral Data of Known Compounds

Further information about the known compounds (**5**–**16**) can be found in Supplementary Materials.

### 3.6. Cytotoxicity Assay

#### 3.6.1. Cell Culture

Human cancer cell lines: HeLa (cervical), A549 (lungs) and MDA MB321 (breast) were maintained in T75 flasks in DMEM (F12/GlutaMax) medium (Invitrogen, Carlsbad, CA, USA), supplemented with 10% heat-inactivated bovine serum (Gibco, Bigcabin, OK, USA) and 1× penicillin-streptomycin (Gibco) at 37 °C in a humified chamber with 5% CO_2_ supply.

#### 3.6.2. Cytotixicty Assay

Cells were seeded (10^5^ cells/well) in 96-well flat-bottom plates (Becton-Dickinson Labware) a day before treatment and grown overnight. Compounds were dissolved in DMSO (Sigma, Saint Louis, MO, USA) and finally prepared as 1.0 mg/mL and 5.0 mg/mL stocks, respectively in culture media. The final concentration of DMSO never exceeded 0.1% in the treatment doses. Three different doses of compounds (50, 25 and 12.5 μg/mL) were further prepared by diluting the stocks in culture media, and cells were treated (in triplicate/dose). Doxorubicin was included as standard reference drug. The treated cultures were further incubated for 48 h, and cell viability test was performed using TACS MTT Cell Proliferation and Viability Assay Kit (TACS) as per manufacturer’s instructions. The optical density (OD) was recorded at 570 nm in a microplate reader (BioTek, ELx800). The cell survival fraction was calculated as (A − B)/A, where A and B are the OD of untreated and treated cells, respectively. The 50% cell survival (IC_50_) values were estimated using the best fit regression curve method in Excel software.

#### 3.6.3. Microscopy

A direct visual investigation was made under an inverted microscope (40× and 100×, Richter Optica, Carlsbad, CA, USA) to observe any morphological changes in the cells cultured with different treatment doses at 24 and 48 h.

## 4. Conclusions

Sixteen compounds, including three new labdane-type diterpenic acids (**1**–**3**) and one new 3,4-seco-triterpenoic acid (**4**), were isolated from the *n*-hexane and chloroform-soluble fractions of the ethanol extract of the aerial parts of *Nuxia oppositifolia*. Of these, most of the tested compounds showed promising cytotoxic activities against human cancer cells in vitro. The results of our study therefore justify the use of *N. oppositifolia* in traditional medicine.

## Figures and Tables

**Table 1:** ^1^H-NMR spectral data (500 MHz, CDCl_3_) of compounds **1**–**4** ^a^.

Position	1	2	3	4 ^b^
1	1.00 m 2.13 m	1.27 m 2.10 m	2.03 m	1.54 m
2	4.94 t	4.98 t (10.7)	4.90 t (11.0)	2.04 m
3	1.17 1.71	1.18 br d (12.0) 1.80 br d (10.8)	1.14 m 1.61 m	
4				1.71 m
5	1.15 br d (4.4)	1.59 br s	1.97 s	0.96 br d (11.7)
6	1.82 1.90	2.40 br d (16.5) 2.26 m		1.32 m 1.33 m
7	5.34 br. s		5.69 br s	1.37 m
8				
9	1.62		2.03 m	1.71 m
10				
11	1.18 1.35	2.03 m 2.19 m	1.35 m	1.81 m
12	1.20 1.37	1.27 m 1.37 m	1.37 m	5.19 br s
13	1.80 m	1.95 m	1.93 m	
14	2.07 2.28	2.10 m 2.26 m	2.03 m 2.23 m	
15				1.93 dd 0.83 m
16	0.93 d (6.7)	0.95 d (6.2)	0.97 d (6.7)	1.63 m 0.97 m
17	1.62	1.64 s	1.82 s	
18	0.87 s	0.85 s	1.08 s	1.83 dd (13.4, 4.7)
19	0.92 s	0.90 s	1.14 s	1.59 br d (13.4) 1.71 m
20	0.79 s	1.06 s	0.82 s	
21-OAc	2.00	1.97 s	1.96 s	1.77 m 1.28 m
22				1.71 m
23				0.78 d (6.8)
24				0.78 d (6.8)
25				0.86 s
26				0.94 s
27				1.13 s
28				0.74 s
29				1.04 s
30				

^a^ Chemical shifts are in ppm; *J* values in Hz are in parentheses; ^b^ Measured in DMSO.

**Table 2:** ^13^C-NMR spectral data (125 MHz, CDCl_3_) of compounds **1**–**4** **^a^**.

No.	1	2	3	4 ^b^
1	44.2 CH_2_	41.0 CH_2_	43.6 CH_2_	32.8 CH_2_
2	68.9 CH	68.1 CH	67.9 CH	28.1 CH_2_
3	46.9 CH_2_	46.1 CH_2_	47.8 CH_2_	174.8 C
4	34.6 C	34.4 C	33.2 C	24.6 C
5	49.5 CH	49.5 CH	62.5 CH	46.9 CH
6	23.5 CH_2_	34.7CH_2_	199.1 CH_2_	17.7 CH_2_
7	122.1 CH	199.9 C	128.5 CH	31.1 CH_2_
8	134.8 C	130.0 C	158.9 C	39.0 C
9	54.3 CH	167.1 C	56.4 CH	37.2 CH
10	39.1 C	42.0 C	44.4 C	39.5 C
11	24.6 CH_2_	27.0 CH_2_	24.6 CH_2_	23.2 CH_2_
12	38.5 CH_2_	35.2CH_2_	38.9 CH_2_	121.9 CH
13	30.9 CH	31.0 CH	30.6 CH	144.2 C
14	41.8 CH_2_	40.9 CH_2_	41.5 CH_2_	41.6 C
15	179.0 C	177.9 C	177.9 C	26.3 CH_2_
16	19.5 CH_3_	19.3 CH_3_	19.4 CH_3_	25.7 CH_2_
17	22.0 CH_3_	11.3 CH_3_	22.0 CH_3_	31.6 C
18	33.0 CH_3_	32.4 CH_3_	34.0 CH_3_	47.8 CH
19	22.5 CH_3_	22.0 CH_3_	22.6 CH_3_	42.3 CH_2_
20	14.2 CH_3_	19.2 CH_3_	15.4 CH_3_	43.1 C
21	21.5 CH_3_	21.4 CH_3_	21.4 CH_3_	30.6 CH_2_
22-OAc	170.8 C	170.8 C	170.7 C	38.0 CH_2_
23				23.2 CH_3_
24				18.7 CH_3_
25				19.0 CH_3_
26				16.5 CH_3_
27				25.3 CH_3_
28				28.1 CH_3_
29				28.2 CH_3_
30				177.9 C

^a^ Assignments were made using HMQC and HMBC experiments; ^b^ Measured in DMSO.

**Table 3:** IC_50_ (μg/mL) values of isolated compounds from *N. oppositifolia*.

Compound No.	Hela (Cervical)	A549 (Lungs)	MDA (Breast)
**1**	87.34	73.37	74.56
**2**	71.34	87.24	68.16
**3**	57.56	72.15	53.34
**4**	37.6	53.78	42.73
**5**	48.96	88.31	54.6
**8**	29.35	79.23	18.25
**9**	42.0	67.37	39.78
**10**	50.2	65.2	47.76
**11**	32.47	83.24	27.16
**12**	57.89	78.69	69.87
**13**	54.58	72.48	63.41
**14**	36.0	57.63	30.0
**15**	85.72	59.17	65.27
Doxorubicin	70.01	164.46	15.41

Pure compounds: Three concentrations (50, 25 and 12.5 μg/mL); All samples done in triplicate and mean values used to calculate IC_50_.

## Supplementary Materials

Supplementary materials are available online.
