# Supplementary material for: New Cytotoxic Seco-Type Triterpene and Labdane-Type Diterpenes from Nuxia oppositifolia
Source: Molecules. 2017 Mar 2;22(3):389. doi: 10.3390/molecules22030389 (PMC6155346; doi:10.3390/molecules22030389)
Supplement: Supplementary file 1 [file molecules-22-00389-s001.pdf]

New Cytotoxic Seco-Type Triterpene and Labdane-Type Diterpenes from *Nuxia oppositifolia*

Shaza M. Al-Massarani [1], Ali A. El-Gamal [1,2,*], Mohammad K. Parvez [1], Mohammed S. Al-Dosari [1], Mansour S. Al-Said [1], Maged S. Abdel-Kader [3,4] and Omer A. Basudan [1]

Content

Spectral Data of known compounds 2

Supplementary Table 1..3

Spectral Data of known compounds

3-Oxolup-20(29)-en-30-al (3-oxolupenal) (**5**), white powder; ^{13}C-NMR data, see Table S1; HR-EI-MS *m/z* = 438.3685 calc. for $C_{30}H_{46}O_2$, 438.3498.

β-sitosterol (**6**), white powder; ^{13}C-NMR data: see Table S1; HR-EI-MS *m/z* = 414.39 calc. for $C_{29}H_{50}O$, 414.7067.

Stigmasterol (**7**), white powder; ^{1}H-and ^{13}C-NMR data: see Table S1; HR-EI-MS *m/z* = 412.36 calc. for $C_{29}H_{48}O$, 412.3705.

3-Oxoolean-12-en-29α–oic acid (katononic acid) (**8**), white powder; ^{13}C-NMR data: see Table S1, HR-EI-MS *m/z* = 454.3447 calc. for $C_{30}H_{46}O_3$, 454.6844.

3-Oxours-12-en-30α-oic acid (ifflaionic acid) (**9**), white powder; ^{13}C-NMR data: see Table S1; HR-EI-MS *m/z* = 454.3447 calc. for $C_{30}H_{46}O_3$, 454.6844.

Ursolic acid (**10**), white powder; ^{1}H-and ^{13}C-NMR data: see Table S1; HR-EI-MS *m/z* = 456.71 calc. for $C_{30}H_{48}O_3$, 456.3603.

30-Hydroxylup-20(29)-en-3-one (3-oxolupenol) (**11**), solid; ^{1}H-and ^{13}C-NMR data: see Table S1; HR-EI-MS *m/z* = 440.70 calc. for $C_{30}H_{48}O_2$, 440.3654.

2α,3β- Dihydroxyolean-12-ene-28-oic acid (maslinic acid) (**12**), solid; ^{13}C-NMR data: see Table S1; HR-EI-MS *m/z* = 472.37 calc. for $C_{30}H_{48}O_4$, 472.3553.

2α-Hydroxy-ursolic acid (asiatic acid) (**13**), solid; ^{13}C-NMR data: see Table S1; HR-EI-MS *m/z* = 488.70 calc. for $C_{30}H_{48}O_5$, 488.7022.

3-Hydroxyurs-12-en-30-oic acid (plectranthoic acid A) (**14**), solid; ^{13}C-NMR data: see Table S1; HR-EI-MS *m/z* = 456.70 calc. for $C_{30}H_{48}O_3$, 456.3603.

Oleanolic acid (**15**), white powder ^{13}C-NMR see Table S1, HR-EI-MS *m/z* = 456.70 calc. for $C_{30}H_{48}O_3$, 456.3603

3,11-Dioxoolean-12-en-28-oic acid (**16**), white powder; ^{1}H-and ^{13}C-NMR data: see Table S1, HR-EI-MS *m/z* = 468.32 calc. for $C_{30}H_{44}O_4$, 468.32.

Table. S1 (^{13}C-NMR, 125 MHz for compounds **5-16**, **5-8** and **15** were measured in $CDCl_3$, while **9-14** and **16** were measured in DMSO-d_6)

#	**5**	**6**	**7**	**8**	**9**	**10**	**11**
1	39.5 CH_2	37.2 CH_2	37.3 CH_2	39.3 CH_2	38.7 CH_2	38.2 CH_2	38.8 CH_2
2	34.0 CH_2	31.6 CH_2	31.8 CH_2	34.2 CH_2	33.6 CH_2	26.9 CH_2	33.6 CH_2
3	217.6 C	71.7 CH	71.8 CH	217.6 C	216.0 C	76.8 CH	216.5 C
4	47.2 C	42.2 CH_2	42.3 CH_2	47.5 C	46.6 C	46.8 C	46.5 C
5	54.8 CH	140.8 C	140.8 C	55.3 CH	54.2 CH	54.7 CH	53.8 CH
6	19.6 CH_2	121.6 CH_2	121.7 CH	19.6 CH_2	19.1 CH_2	18.0 CH_2	19.1 CH_2
7	33.5 CH_2	31.6 CH_2	31.7 CH_2	32.2 CH_2	31.8 CH_2	32.7 CH_2	33.0 CH_2
8	40.6 C	31.8 CH	31.9 CH	39.7 C	39.8 C	39.1 C	40.2 C
9	49.5 CH	50.0 CH	50.1 CH	48.0 CH	36.0 CH	47.0 CH	48.9 CH
10	36.7 C	36.2 C	36.2 C	36.7 C	38.8 C	36.5 C	36.3 C
11	21.4 CH_2	21.1 CH_2	21.1 CH_2	23.6 CH_2	23.1 CH_2	23.7 CH_2	21.0 CH_2
12	27.5 CH_2	39.6 CH_2	39.7 CH_2	122.5 CH	124.4 CH	124.5 CH	25.9 CH_2
13	37.7 CH	42.1 C	42.3 C	144.3 C	138.2 C	138.1 CH	37.6 CH
14	42.7 C	56.8 CH	56.8 CH	41.6 C	41.6 C	41.6 C	42.4 C
15	27.5 CH_2	24.3 CH_2	24.3 CH_2	26.1 CH_2	25.1 CH_2	27.5 CH_2	27.0 CH_2
16	35.2 CH_2	28.8 CH_2	28.3 CH_2	26.9 CH_2	26.0 CH_2	22.8 CH_2	34.9 CH_2
17	43.2 C	55.8 CH	56.1 CH	32.0 C	33.1 C	47.0 C	42.5 C
18	51.0 CH	12.2 CH_3	11.9 CH_3	47.0 CH	57.1 CH	52.3 CH	47.9 CH
19	51.0 CH	19.9 CH_3	19.4 CH_3	42.5 CH_2	33.9 CH	38.4 CH	43.8 CH
20	157.2 C	40.7 CH	36.5 CH	44.1 C	51.6 CH	38.5 CH	155.0 C
21	32.7 CH_2	21.0 CH_3	19.1 CH_3	31.1 CH_2	31.8 CH_2	30.1 CH_2	31.1 CH_2
22	39.8 CH_2	138.2 CH	34.0 CH_2	38.3 CH_2	40.0 CH_2	36.3 CH_2	39.3 CH_2
23	26.6 CH_3	129.5 CH	26.2 CH_2	26.4 CH_3	26.2 CH_3	28.2 CH_3	26.3 CH_3
24	21.0 CH_3	51.2 CH	45.9 CH	21.5 CH_3	21.1 CH_3	16.9 CH_3	20.7 CH_3
25	15.6 CH_3	32.0 CH	29.2 CH	15.2 CH_3	15.0 CH_3	16.0 CH_3	15.6 CH_3
26	16.0 CH_3	21.2 CH_3	19.8 CH_3	16.7 CH_3	16.4 CH_3	15.2 CH_3	15.4 CH_3
27	14.3 CH_3	19.0 CH_3	18.8 CH_3	25.9 CH_3	23.0 CH_3	23.2 CH_3	14.2 CH_3
28	17.8 CH_3	25.5 CH_2	23.1 CH_3	28.2 CH_3	28.0 CH_3	178.3 C	17.4 CH_3
29	133.2 CH_2	12.3 CH_3	12.0 CH_3	28.7 CH_3	18.4 CH_3	17.0 CH_3	105.6 CH_2
30	194.8 C	---	---	183.9	177.1 C	21.0 CH_3	62.5 CH_2

Table. S1 Cont.

#	**12**	**13**	**14**	**15**	**16**
1	46.7 CH_2	47.0 CH_2	39.9 CH_2	38.5 CH_2	39.7 CH_2
2	67.1 CH	67.1 CH	27.0 CH_2	27.3 CH_2	34.2 CH_2
3	82.2 CH	82.2 CH	76.8 CH	79.2 CH	217.4 C
4	39.9 C	39.2 C	39.3 C	38.5 C	47.8 C
5	54.7 CH	54.7 CH	54.7 CH	55.3 CH	55.4 CH_2
6	18.0 CH_2	18.0 CH_2	17.9 CH_2	18.4 CH_2	18.8 CH_2
7	32.3 CH_2	32.6 CH_2	32.4 CH_2	32.7 CH_2	32.1 CH_2
8	38.9 C	38.9 C	39.1 C	39.4 C	43.8 C
9	47.0 CH	46.9 CH	47.0 CH	47.8 CH	61.1 CH
10	37.6 C	37.5 C	36.4 C	37.2 C	36.7 C
11	22.9 CH_2	22.9 CH_2	22.9 CH_2	23.0 CH_2	199.8 C
12	121.4 CH	124.4 CH	124.5 CH	122.8 CH	128.4 CH
13	143.8 C	138.2 C	138.2 C	143.7 C	170.0 C
14	41.3 C	41.6 C	41.5 C	41.7 C	43.3 C
15	27.1 CH_2	27.5 CH_2	27.3 CH_2	27.8 CH_2	26.4 CH_2
16	23.0 CH_2	22.9 CH_2	25.1 CH_2	23.5 CH_2	26.5 CH_2
17	45.4 C	46.9 C	33.1 C	46.7 C	45.3 C
18	40.7 CH	52.3 CH	57.0 CH	41.1 CH	48.2 CH
19	45.7 CH_2	40.7 CH	33.9 CH	46.0 CH_2	40.9 CH_2
20	30.3 C	38.4 CH_2	51.6 CH	30.8 C	31.9 C
21	33.3 CH_2	30.2 CH_2	32.4 CH_2	33.9 CH_2	37.7 CH_2
22	32.3 CH_2	36.3 CH_2	38.3 CH_2	32.6 CH_2	34.2 CH_2
23	28.7 CH_3	28.8 CH_3	28.2 CH_3	28.2 CH_3	26.4 CH_3
24	16.8 CH_3	16.9 CH_3	16.5 CH_3	15.7 CH_3	21.5 CH_3
25	16.3 CH_3	16.4 CH_3	16.0 CH_3	15.5 CH_3	15.7 CH_3
26	17.0 CH_3	17.1 CH_3	15.4 CH_3	17.2 CH_3	18.5 CH_3
27	25.6 CH_3	25.4 CH_3	23.1 CH_3	26.1 CH_3	23.4 CH_3
28	178.6 C	178.6 C	28.0 CH_3	183.9 C	182.0 C
29	32.6 CH_3	23.3 CH_3	18.4 CH_3	33.2 CH_3	28.6 CH_3
30	22.6 CH_3	21.1 CH_3	177.1 C	23.7 CH_3	28.5 CH_3
